# Real-World Experience of Risankizumab in Refractory Pediatric Inflammatory Bowel Disease

**DOI:** 10.14309/crj.0000000000002316

**Published:** 2026-09-29

**Authors:** Ali Alsarhan, Balaji Krishnamurthy, Eman Elyyan, Mohammad Alhajjar, Christos Tzivinikos

**Affiliations:** 1 Department of Pediatric Gastroenterology, Al-Jalila Children's Hospital, Dubai, United Arab Emirates; 2 Clinical Pharmacy Section, Al-Jalila Children's Hospital, Dubai, United Arab Emirates

**Keywords:** pediatric inflammatory bowel disease, risankizumab, intestinal ultrasound

## Abstract

The incidence of pediatric inflammatory bowel disease (IBD) is rising globally. Anti-tumor necrosis factor agents remain the mainstay of treatment, but a significant number of patients experience treatment failure. This case-series presents real-world experience using risankizumab, an IL-23 inhibitor, in 9 children with severe-refractory IBD. Multiple biologics had failed in all patients before initiation. Response was assessed using symptoms, biomarkers, endoscopy, and imaging. Five patients achieved clinical remission, and 4 failed to achieve complete remission. Noninvasive monitoring tools, such as intestinal ultrasound, were used. No adverse events were reported. Risankizumab may be a promising option in refractory pediatric IBD.

## INTRODUCTION

The global incidence and prevalence of pediatric inflammatory bowel disease (IBD) are rising, with increasing disease severity and complexity. Cases are now emerging in regions where IBD was previously rare, indicating an epidemiological shift.^1^

Therapeutic options for pediatric IBD are limited, with anti-TNF agents such as infliximab and adalimumab being the only approved biologics. Treatment failure—whether due to pharmacodynamic or pharmacokinetic loss of response—remains a serious challenge. Annual discontinuation of infliximab due to loss of response has been reported at a rate of 3.2% per year.^2^ Adalimumab is also used widely but its effectiveness is limited, with up to 55% of pediatric patients experiencing treatment failure within the first year.^3^

Newer therapies like vedolizumab and ustekinumab are being used in pediatric Crohn's disease, but with variable effectiveness. Vedolizumab achieved clinical remission in only 25% of patients at 1 year,^4^ while ustekinumab showed limited success, with 28% experiencing drug failure and just 58% attaining steroid-free remission.^5^ These findings underscore the therapeutic ceiling of current biologics and highlight the need for evidence-based sequencing to optimize second-line and third-line treatments.

Risankizumab, a selective IL-23 inhibitor targeting the p19 subunit, has been in clinical use since 2017. This humanized IgG monoclonal antibody was initially approved for moderate-to-severe plaque psoriasis, where it demonstrated strong efficacy.^6^ Subsequently, risankizumab was studied in the ADVANCE clinical trial for moderate-to-severe Crohn's disease, which included only small number of patients aged between 16 and 18 years. These trials demonstrated that the standard dosing regimen (600 mg IV at weeks 0, 4, and 8) achieved clinical remission in 45% of patients and endoscopic remission in 40%.^7^ Risankizumab also shows clear efficacy in ulcerative colitis, with week-12 remission 20.3% vs 6.2% on placebo and week-52 remission 40.2% (180 mg) and 37.6% (360 mg) vs 25.1% after placebo withdrawal, with no major safety concerns.^8^

There are currently no published data on risankizumab in pediatric Crohn's disease. This case series offers early real-world insights into its use in refractory pediatric IBD.

### Case #1

A 15.5-year-old adolescent girl was diagnosed with Crohn's disease at age of 13. She had proximal and distal ileal disease with stricture and intra-abdominal abscess. She required ileocecal resection for intestinal obstruction, and to prevent recurrence, she received adalimumab and started ‘Crohn's disease exclusion diet’. After 2 years, she developed pharmacodynamic loss of response and magnetic resonance enterography (MRE) showed active disease of proximal ileum. Ustekinumab was trialed for 5 months (4 weekly dosing) with systemic steroid tapering course, but MRE still showed active disease with high C-reactive protein (CRP) and erythrocyte sedimentation rate (ESR), prompting initiation of risankizumab. Despite being asymptomatic, at 12 weeks ESR rose to 88 mm/hr and CRP remained elevated (12.9 mg/dL); repeat MRE confirmed ongoing disease activity. After 7 months, daily dose of upadacitinib 30 mg was added for total of 3 months without normalization of ESR (45 mm/hr), so it was discontinued, and risankizumab 360 mg was intensified to every 4 weeks. After 6 months of intensified dosing, enteroscopy, capsule endoscopy, and MRE demonstrated complete mucosal healing. The dose of risankizumab was gradually spaced out to 8-weekly, and she sustained clinical remission.

### Case #2

A 16-year-old adolescent boy, presented with diarrhea and was diagnosed with Crohn's disease at age 14. He had ileocolonic, gastric, and duodenal distribution of the disease. He required ileostomy due to perforation during colonoscopy (reversed after a period of stabilization) and was treated with adalimumab for 2 years before developing pharmacodynamic loss of response. MRE showed active disease of distal ileum, leading to initiation of risankizumab 2 years after diagnosis. He remained clinically asymptomatic with normal inflammatory markers. Intestinal ultrasound (IUS) at 3 months was normal, and repeat IUS at 16 months from initiation of risankizumab confirmed normal bowel wall thickness (<3 mm) of terminal ileum and colon.

### Case #3

A 15-year-old adolescent boy was diagnosed with Crohn's disease at age 11 years after presenting with abdominal pain, diarrhea, and weight loss, and had ileocolonic disease with ileal stricture. Initial induction with infliximab and tapering steroids was effective, but pharmacokinetic loss of response developed after 2 years. Adalimumab, combined with azathioprine, was used for 2 years before pharmacodynamic loss of response occurred. Endoscopy revealed ulceration in the stomach, rectosigmoid, and terminal ileum, while MRE confirmed active ileal inflammation with stenosis, prompting initiation of risankizumab. At initiation, he was asymptomatic but had elevated CRP (12.8 mg/dL) and ESR (27 mm/hr). Over 2 years of follow-up, he remained clinically well with normalization of inflammatory markers, hemoglobin improved from 9.8 to 13.6 g/dL, and platelets decreased from 395 to 277. IUS showed normal bowel wall thickness (<3 mm) of terminal ileum and colon.

### Case #4

A 12-year-old adolescent girl presented with severe diarrhea and was diagnosed with Crohn's disease at age 10. She had colonic disease with strictures. Adalimumab was used for 2 years but developed pharmacodynamic loss of response. She received topical budesonide for 8 weeks before starting risankizumab along with Crohns' Disease Exclusion Diet. At initiation, she had severe diarrhea, pyoderma gangrenosum and active terminal ileum, and cecal disease on MRE. Over 2 years of follow-up, she achieved clinical remission, disappearance of pyoderma gangrenosum, ESR decreased from 51 to 15 mm/hr, hemoglobin slightly improved (9.6 → 9.9 g/dL), and IUS showed normal bowel wall thickness (<3 mm) of terminal ileum and colon.

### Case #5

A 16-year-old adolescent girl, diagnosed with Crohn's disease at age 14 after presenting with abdominal pain, diarrhea, and weight loss, also had sickle cell anemia requiring multiple blood transfusions. She had ileocolonic disease with a perianal fistula. Previous therapies included infliximab (7 months), adalimumab (3 months), and vedolizumab (4 months), all discontinued due to pharmacokinetic loss of response with anti-drug antibodies formation. Methotrexate and azathioprine were trialed but stopped due to adverse events. Risankizumab was initiated nearly 2 years after diagnosis; however, she remained symptomatic with abdominal pain and elevated inflammatory markers (CRP 22.9 mg/dL, ESR 96 mm/hr). After 6 months, there was no symptomatic improvement, persistent ED visits for abdominal pain and diarrhea, and inflammatory markers remained high despite dose escalation and reinduction as shown in Table 1. Repeat endoscopy confirmed active disease. Of note, intestinal ultrasound (IUS) performed after 2 doses of 1,200 mg demonstrated a reduction in bowel wall thickness of the affected colonic segment from 8 mm to 5–6 mm. She was referred for bone marrow transplant but parents refused.

**Table 1.** Patient's characteristics, previous treatments, and outcomes in pediatric Crohn's disease cohort treated with risankizumab

No.	Gender	Age at commencing risankizumab	Disease	Paris classification	Previous biological medication used and duration	Concurrent use of other drugs with risankizumab	Dose of risankizumab	Successful induction at 12 weeks	Follow-up modality	Duration of follow-up	Final response
#1	Female	15 years	Crohn's disease	A1b, L3, B2B3	Adalimumab, ustekinumab	No	600 mg IV at 0,4,8, then SC 360 mg at 12, then 360 mg every 8 weeks. Transient escalation to every 4 weeks dosing for 6 months.	No	MRE and capsule enteroscopy	30 months	Remission
#2	Male	16 years	Crohn's disease	A1b, L3+L4a, B1	Adalimumab	No	600 mg IV at 0,4,8, then SC 360 mg at 12, then 360 mg every 8 weeks.	Yes (normal bowel ultrasound)	Bowel ultrasound	25 months	Remission
#3	Male	15 years	Crohn's disease	A1b, L3, B2	Infliximab	No	600 mg IV at 0,4,8, then SC 360 mg at 12, then 360 mg every 8 weeks.	Yes (normal bowel ultrasound), normalized CRP and ESR	Colonoscopy	25 months	Active disease
#4	Female	12 years	Crohn's disease	A1b, L2, B2	Adalimumab	Topical (budesonide for 8 weeks)	600 mg IV at 0,4,8, then SC 360 mg at 12, then 360 mg every 8 weeks.	Yes (normal bowel ultrasound), improved symptoms	Resolution of symptoms	25 months	Remission
#5	Female	16 years	Crohn's disease	A1b, L3, B3, p	Infliximab, adalimumab, vedolizumab	No	600 mg IV at 0,4,8, then SC 360 mg at 12, then 360 mg every 8 weeks. Re-induction with 1,200 mg at 0,4,8 weeks.	No	Colonoscopy and bowel ultrasound	10 months	No remission
#6	Female	13 years	Crohn's disease	A1b, L1, B1	Infliximab, adalimumab, ustekinumab	Topical (budesonide for 8 weeks)	600 mg IV at 0,4,8, then SC 360 mg at 12, then 360 mg every 8 weeks.	Yes (normal bowel ultrasound), improved symptoms	Colonoscopy and bowel ultrasound	13 months	Remission
#7	Female	13 years	Crohn's disease	A1b, L3, B1	Infliximab, adalimumab	No	600 mg IV at 0,4,8, then medication was discontinued.	Yes (symptoms improved)	Colonoscopy and bowel ultrasound	7 months	Remission
#8	Male	9 years	Ulcerative colitis	E2, S1	Infliximab, vedolizumab, dual (infliximab + vedolizumab), dual (vedolizumab + tacrolimus)	Infliximab	600 mg IV at 0,4,8, then SC 360 mg at 12, then 360 mg every 8 weeks.	Yes (symptoms improved)	Colonoscopy and persistent high ESR	8 months	Active disease
#9	Male	17 years	Ulcerative colitis	E4, S0	Vedolizumab	Topical (budesonide for 8 weeks)	600 mg IV at 0,4,8, then re-induction 1,200 mg at 0,4,8 weeks (due to poor response) then 360 mg SC afterward.	Yes (symptoms improved)	Colonoscopy and bowel ultrasound	7 months	Active disease

CRP, C-reactive protein; ESR, erythrocyte sedimentation rate; SC, subcutaneous.

### Case #6

A 13-year-old adolescent girl was diagnosed with Crohn's disease involving the terminal ileum at age of 11. She discontinued infliximab due to a severe reaction and high anti-drug antibody titers. Adalimumab was used for 7 months but stopped due to skin lesions concerning for neoplasm. Ustekinumab was trialed for 7 months without achieving remission despite high drug levels (>5 μg/mL). She also received steroids, azathioprine, and topical budesonide at different times. Risankizumab was initiated when she was symptomatic with abdominal pain, elevated fecal calprotectin (1800 μg/g), Colonic wall thickening on ultrasound (5 mm), and active terminal ileum disease on MRE. Over 6 months, she showed resolution of symptoms with normalization of terminal ileum thickness (<3 mm) on IUS.

### Case #7

A 13-year-old adolescent girl presented with abdominal pain, diarrhea, and weight loss, initially diagnosed with ulcerative colitis (pancolitis); subsequent identification of terminal ileum involvement led to revision of the diagnosis to Crohn's disease. She was treated with infliximab for 8 months and later adalimumab for 2 years, both discontinued due to pharmacokinetic loss of response. Then, she received 3 induction doses of risankizumab, after which therapy was stopped by her father for nonclinical reasons. At 6-month follow-up off treatment, she remained asymptomatic, with completely normal endoscopic findings and persistently normal laboratory results.

### Case #8

A 3-year-old boy diagnosed with severe very-early-onset IBD with predominantly rectosigmoid involvement had multiple therapy failures, including infliximab, vedolizumab, advanced combination of infliximab plus vedolizumab, and even vedolizumab plus tacrolimus. He had persistent intermittent bloody diarrhea, suboptimal weight gain, fecal calprotectin >800 μg/g, and ESR 30 mm/h. Risankizumab was initiated with discontinuation of tacrolimus and infliximab (due to neutralizing antibodies and low drug level which led to pharmacokinetic loss of response). After 8 months, his symptoms improved, although ESR remained elevated at 28 mm/hr; IUS was normal both before and after treatment and no follow-up endoscopy has been performed as parents preferred noninvasive monitoring. He developed recurrent skin abscesses requiring drainage after starting risankizumab, but previous herpetic stomatitis on infliximab makes causality uncertain.

### Case #9

A 17-year-old adolescent boy with ulcerative colitis (pancolitis) was initially treated with systemic steroids and 5-ASA, followed by vedolizumab; 5-ASA was discontinued after he developed pancreatitis, and he had no response to 5 months of 300 mg vedolizumab every 4 weeks. He continued to have active disease with Pediatric Ulcerative Colitis Activity Index (PUCAI) 30, pancolitis on endoscopy, and intestinal ultrasound showing sigmoid and descending colon wall thickening (4 mm), despite normal laboratory results. Risankizumab was initiated at 600 mg IV at weeks 0, 4, and 8 in addition to topical steroids; due to poor response, reinduction was given of 1,200 mg IV at weeks 12, 16, and 20 followed by 360 mg subcutaneous maintenance. After 7 months of follow-up from initiation of risankizumab, his symptoms improved and PUCAI decreased from 30 to 5; however, intestinal ultrasound continued to show persistent sigmoid and descending colon thickening (4 mm), and endoscopy demonstrated ongoing active disease. Owing to that, dosing was changed to every 4 weeks.

## DISCUSSION

Although risankizumab is approved for adults, no pediatric data have been published. This case series explores its use in severe pediatric IBD and reports early safety outcomes.

All patients in our case series (N = 9) had previously received at least 1 biological therapy, which failed to control disease activity as evidenced by either clinical symptoms, biochemical markers, Endoscopic evaluation or imaging (Table 1). Additional biologics, including ustekinumab and vedolizumab, were also unsuccessful. Disease activity was confirmed by MRE in 5 patients and by endoscopy with biopsy in 3. At the time of risankizumab initiation, 6 out of 9 patients had active symptoms.

Cases #4,#6, and #9 received topical oral budesonide during risankizumab induction, case #8 was on infliximab. No patients were treated with systemic corticosteroids or immunomodulators concurrently or afterward. Case #1 did not respond to standard risankizumab or combination therapy with upadacitinib; however, switching to 4-weekly risankizumab led to full remission, confirmed by endoscopy and MRE.^9^ Cases #5 and #9 underwent dose escalation with high-dose reinduction (1,200 mg) but it failed to respond, with colonoscopy showing persistent active inflammation. This indicates that while reinduction and dose escalation can help some patients unresponsive to standard regimens, they are not universally effective and may still fail.

### Induction of therapy and sustaining remission

Therapeutic response was assessed 12 weeks after the first dose using symptoms, CRP, ESR, and intestinal ultrasound. Seven patients improved symptomatically, with 4 showing normal bowel thickness (<3 mm) on ultrasound. One patient who failed induction continued to do so, while another nonresponder achieved remission after dose escalation. Three patients who initially responded clinically/biochemically still had active disease on colonoscopy. This shows that symptom and ultrasound improvement does not always reflect mucosal remission, and laboratory markers can be misleading. On the other hand, Case #7 received 3 induction doses before the parents discontinued therapy. The patient achieved remission, which was maintained for at least 6 months and confirmed by endoscopy. This suggests that the medication was able to induce remission that was sustained even after discontinuation.

### Side effects

Patients were followed for a period ranging from 7 months to 30 months after initiating risankizumab. No adverse effects were reported during follow-up. Case #8 developed recurrent skin abscesses requiring drainage, but the patient had a history of previous skin infections and had also experienced herpetic lesions previously.

### Intestinal ultrasound as non invasive monitoring tool

Seven patients were monitored using intestinal ultrasound. IUS has shown good correlation with disease activity in pediatric IBD and is increasingly used as a noninvasive monitoring tool.^10^ IUS has been useful for comparing bowel wall thickness before and after treatment and for monitoring patients with normal thickness to detect early disease activity. In this cohort, many parents preferred ultrasound because it is noninvasive, did not require anesthesia, and could be performed quickly at the bedside while providing valuable clinical activity information. Nonetheless, it should not be used in isolation—treatment decisions must consider all clinical, laboratory, endoscopic, and imaging findings together.

Our case series provides early real-world data supporting the potential role of risankizumab as a treatment in children with severe refractory IBD. Despite previous failure of multiple biologics, several patients could achieve remission without adverse effects. While these findings are promising, larger prospective studies and long-term follow-up are essential to validate risankizumab's role and optimize its use in pediatric IBD management.

## DISCLOSURES

Author contributions: A. Alsarhan: Conceptualized the study, conducted the literature review, collected the data drafted the entire manuscript, created the table, and performed the final review of the article. B. Krishnamurthy, E. Elyyan: Contributed to writing and reviewing the manuscript. M. Alhajjar: Assisted in writing and critically reviewed the manuscript. C. Tzivinikos: Supervised the project and critically reviewed the entire manuscript. All authors approved the final version of the manuscript. C. Tzivinikos is the article guarantor.

Acknowledgments: We thank the pharmacy department for ensuring the availability of the medication, and the nursing staff for their invaluable support in administering the treatment.

Financial disclosure: None to report.

Informed consent was obtained for this case report.

## ABBREVIATIONS:

anti-TNF: anti-Tumor Necrosis Factor
CDED: Crohns' Disease Exclusion Diet
CRP: C reactive protein
ESR: Erythrocyte Sedimentation Rate
IBD: Inflammatory Bowel Disease
IL-23: Interleukin-23
IUS: Intestinal Ultrasound
MRE: Magnetic Resonance Enterography
PUCAI: Pediatric Ulcerative Colitis Activity Index
SC: subcutaneous
